# Adrenal suppression: A practical guide to the screening and management of this under-recognized complication of inhaled corticosteroid therapy

**DOI:** 10.1186/1710-1492-7-13

**Published:** 2011-08-25

**Authors:** Alexandra Ahmet, Harold Kim, Sheldon Spier

**Affiliations:** 1 University of Ottawa, Children's Hospital of Eastern Ontario, Ottawa, Ontario, Canada; 2 University of Western Ontario, London, Ontario, Canada; 3 McMaster University, Hamilton, Ontario, Canada; 4 University of Calgary, Alberta Children's Hospital, Calgary, Alberta, Canada

## Abstract

Inhaled corticosteroids (ICSs) are the most effective anti-inflammatory agents available for the treatment of asthma and represent the mainstay of therapy for most patients with the disease. Although these medications are considered safe at low-to-moderate doses, safety concerns with prolonged use of high ICS doses remain; among these concerns is the risk of adrenal suppression (AS). AS is a condition characterized by the inability to produce adequate amounts of the glucocorticoid, cortisol, which is critical during periods of physiological stress. It is a proven, yet under-recognized, complication of most forms of glucocorticoid therapy that can persist for up to 1 year after cessation of corticosteroid treatment. If left unnoticed, AS can lead to significant morbidity and even mortality. More than 60 recent cases of AS have been described in the literature and almost all cases have involved children being treated with ≥500 μg/day of fluticasone.

The risk for AS can be minimized through increased awareness and early recognition of at-risk patients, regular patient follow-up to ensure that the lowest effective ICS doses are being utilized to control asthma symptoms, and by choosing an ICS medication with minimal adrenal effects. Screening for AS should be considered in any child with symptoms of AS, children using high ICS doses, or those with a history of prolonged oral corticosteroid use. Cases of AS should be managed in consultation with a pediatric endocrinologist whenever possible. In patients with proven AS, stress steroid dosing during times of illness or surgery is needed to simulate the protective endogenous elevations in cortisol levels that occur with physiological stress.

This article provides an overview of current literature on AS as well as practical recommendations for the prevention, screening and management of this serious complication of ICS therapy.

## Background

Asthma is the most common chronic disease among the young, affecting 10% to 15% of Canadian children and adolescents [1-3]. It is also a major cause of pediatric hospital admissions and emergency department visits [4,5]. Despite significant improvements in the diagnosis and management of asthma over the past decade, as well as the availability of comprehensive and widely-accepted national and international clinical practice guidelines for the disease [6,7], asthma control in Canada remains suboptimal. Approximately 50-60% of Canadian children and adults have uncontrolled disease according to guideline-based asthma control criteria [8,9].

Inhaled corticosteroids (ICSs) are the most effective anti-inflammatory medications available for the treatment of asthma and represent the mainstay of therapy for most patients with the disease. The current Canadian standard of care is low-dose ICS monotherapy as first-line maintenance therapy for most children and adults with asthma [7]. Regular ICS use has been shown to reduce symptoms and the need for rescue beta-agonists, prevent exacerbations, improve lung function and quality of life, and reduce hospitalizations and asthma-related mortality [6,7,10-13].

In children of any age, ICS starting doses are similar to those recommended in adults (see Table 1) [14]. At low-to-moderate doses, ICSs are considered safe medications, and are generally not associated with clinically significant adverse effects. Furthermore, studies have shown that ICS treatment markedly reduces the need for oral corticosteroids, which have been associated with well-known serious adverse effects [15].

**Table 1:** ICS starting doses for asthma therapy in children in Canada

ICS and inhaler device	Minimum age licensed for use	Low-moderate dose (μg/day)	High-dose (μg/day)
Beclomethasone dipropionate MDI (Qvar, generics)	5 years	100-150 BID	200 BID

Budesonide DPI (Pulmicort)	6 years	200 BID	400 BID

Budesonide Nebulizer (Pulmicort)	3 months	250-500 BID	1000 BID

Ciclesonide MDI (Alvesco)	6 years	100-200 OD	400 OD-BID

Fluticasone propionate MDI/DPI (Flovent HFA, Flovent Diskus)	12 months	100-125 BID	250 BID

ICS: inhaled corticosteroid; MDI: metered dose inhaler; DPI: dry powder inhaler; BID: twice daily; OD: once daily

Adapted from Kovesi et al., 2010 [14]

Although the side effects of ICSs are less frequent and severe than those of oral corticosteroids, safety concerns with these agents still remain, particularly when used at high doses. Among these concerns is the risk of adrenal suppression (AS) -- a condition characterized by the inability to produce adequate amounts of cortisol (a glucocorticoid that is critical during periods of physiological stress). AS is an under-recognized complication of ICS therapy that, if left unnoticed, can lead to significant morbidity and even mortality [16-19]. The purpose of this review is to assist physicians and other healthcare professionals in identifying patients who may be at risk for AS, and provide practical recommendations for the screening and management of this potentially serious side effect of ICS therapy.

## Adrenal Suppression (AS): Definition, Pathophysiology and Clinical Presentation

### Definition

Adrenal insufficiency is a condition in which the adrenal glands are unable to produce adequate amounts of cortisol (a glucocorticoid responsible for maintaining blood pressure, blood glucose and energy levels during times of physiological stress, such as illness, surgery or injury). It can result from any etiology (i.e., genetic, iatrogenic, acquired), and may also be associated with other adrenal hormone deficiencies, such as impaired aldosterone production (see Table 2) [20].

**Table 2:** Adrenal insufficiency, adrenal suppression (AS), and adrenal crisis: definitions and symptoms [20,21]

Definition	Signs/Symptoms
**Adrenal insufficiency**: Adrenal glands unable to produce a sufficient amount of cortisol secondary to ANY etiology (genetic, iatrogenic, acquired); may be associated with other adrenal hormone deficiencies.	► Weakness/fatigue ► Malaise ► Nausea ► Vomiting ► Diarrhea ► Abdominal pain ► Headache (usually in the morning) ► Poor weight gain ► Poor linear growth ► Myalgia ► Arthralgia ► Psychiatric symptoms

**Adrenal suppression (AS)**: Adrenal glands unable to produce a sufficient amount of cortisol secondary to exposure of the HPA axis to exogenous glucocorticoids, leading to suppression and, in turn, adrenal insufficiency.	► Weakness/fatigue ► Malaise ► Nausea ► Vomiting ► Diarrhea ► Abdominal pain ► Headache (usually in the morning) ► Poor weight gain ► Poor linear growth ► Myalgia ► Arthralgia ► Psychiatric symptoms

**Adrenal crisis**: Severe, life-threatening adrenal insufficiency; can occur with AS.	► Hypotension ► Hypoglycemia (seizure, coma)

HPA: hypothalamic-pituitary-adrenal

AS is the most common cause of adrenal insufficiency, and refers to decreased or inadequate cortisol production that results from exposure of the hypothalamic-pituitary-adrenal (HPA) axis to exogenous glucocorticoids (see Table 2) [20,21]. It is a proven, yet under-recognized, complication of most forms of glucocorticoid therapy (e.g., inhaled, oral, intramuscular, intranasal, intravenous) that can persist for up to 1 year after cessation of corticosteroid treatment [16,22]. More than 60 recent cases of AS have been described in the literature [22,23]. Although the risk factors for the development of this condition have not been clearly established, increased dose and longer duration of glucocorticoid therapy appear to be associated with an increased risk [22]. In fact, the Pediatric Endocrine Society suggests that AS be considered in all children who have received supraphysiological doses of oral corticosteroids (>8-12 mg/m^2^/day - hydrocortisone equivalent) for greater than 2 weeks [24]. AS is also considered to be an important risk in children who require long-term treatment with high-dose ICS therapy. Children who are being treated for asthma often receive other forms of glucocorticoids in addition to ICSs (i.e., intranasal, oral, intravenous) and, therefore, the patients' "total steroid load" must be considered when evaluating the risk of AS.

If AS is left unrecognized and the body is subjected to physiological stress, such as injury, surgery or a severe infection, the condition can lead to an adrenal crisis (see Table 2). Adrenal crisis is defined as severe, life-threatening adrenal insufficiency characterized by severe hypotension and/or hypoglycemia which may lead to seizures and even coma [20,21,25-28]. Although it is considered a rare consequence of AS, a retrospective survey in the United Kingdom (UK) found that the frequency of acute adrenal crisis in children using ICS therapy was greater than previously expected [17].

### Pathophysiology

The HPA axis is under circadian regulation and operates in a negative feedback loop to regulate cortisol secretion within the body. The hypothalamus releases corticotropin-releasing hormone (CRH), with peak levels being produced in the morning (around 6 am). CRH then stimulates the release of adrenocorticotropic hormone (ACTH) from the pituitary gland, which, in turn, stimulates the adrenal glands to secrete cortisol. Cortisol has inhibitory effects on the hypothalamus and pituitary gland, which leads to decreased secretion of CRH and ACTH and, in turn, reduced production and secretion of cortisol. This negative-feedback loop allows the HPA axis to tightly self-regulate cortisol levels in the body. Exogenous glucocorticoids exert negative feedback in the same manner as endogenous cortisol, leading to the suppression of cortisol production and, subsequently, adrenal insufficiency [29]. Since cortisol production is critical during periods of physiological stress (i.e., illness or surgery), its suppression by exogenous glucocorticoids can lead to significant morbidity (adrenal crisis) and even mortality.

### Clinical Presentation

The clinical presentation of AS is highly variable. Symptoms are often non-specific and may include: weakness, fatigue, malaise, nausea, abdominal pain, poor weight gain, and headache (see Table 2). In some cases, AS may be associated with biochemical changes in the absence of symptoms [21]. Decreased growth may also be a clinical sign of AS and is often seen in children with significant AS. However, growth suppression may also be a primary side effect of ICS therapy or may occur secondary to poor asthma control [30-34]. Therefore, decreased growth is neither a sensitive nor a specific indicator of AS [35].

Given the non-specific nature of the symptoms of AS, the disorder can often go unrecognized until physiologic stress (e.g., simple gastroenteritis, minor upper respiratory tract infection, surgery) precipitates an adrenal crisis. Many of the symptoms of these common stressors are so similar to those of adrenal suppression that the first signs of AS may go unnoticed, unless there is a high level of suspicion and the families of patients at risk are made aware of the possibility of this side effect. The symptoms of adrenal crisis include: hypotension and unexplained, acute hypoglycemia that often leads to seizures, decreased consciousness, and even coma [21].

In children presenting with symptoms suggestive of AS, it is important to rule out a primary cause of adrenal insufficiency (i.e., Addison's disease). In primary adrenal insufficiency, individuals usually have both symptoms of glucocorticoid deficiency (consistent with AS) as well as symptoms of mineralocorticoid deficiency. Mineralocorticoids stimulate sodium reabsorption and potassium excretion. Symptoms of mineralocorticoid deficiency include salt cravings, volume depletion and weight loss. Unlike AS, adrenal crisis associated with mineralocorticoid deficiency is often associated with hyponatremia and hyperkalemia [36,37]. Findings consistent with mineralocorticoid deficiency should prompt the physician to consider a primary cause of the patient's symptoms.

### Testing

Several endocrine tests have been used for the screening and diagnosis of AS. The insulin-induced hypoglycemia test (IIHT) was once considered the gold standard for the diagnosis of adrenal insufficiency, but is no longer used in children due to the neurocognitive risks associated with hypoglycemia. The standard-dose (250 μg) ACTH stimulation test was previously the best available test; however, a recent meta-analysis found the sensitivity of this test to be suboptimal when compared to the newer, low-dose (1 μg) ACTH stimulation test [38]. Although these findings remain controversial in the medical community, the low-dose ACTH stimulation test is now considered by many to be the best test for diagnosing AS in children. This test involves the intravenous administration of 1 μg of cosyntropin followed by the measurement of serum cortisol levels at baseline (0 min), 15-20 min and 30 min to assess the function of the HPA axis. A peak cortisol level >500 nmol/L is considered a normal response; a peak level <500 nmol/L is diagnostic of AS, with both a sensitivity and specificity of approximately 90% [38-46]. Given the natural circadian variation in cortisol secretion, the test should be performed in the morning to ensure optimal sensitivity and specificity [47].

Although the low-dose ACTH stimulation test is currently the most sensitive and specific test for AS, a first morning (08:00 am) cortisol measurement is often more practical and is considered to be a reasonable first step for the identification of cases of suspected AS, or for the screening of children being treated with high-dose ICS therapy. The specificity of this test approaches 100% if a very low cut-off value (<85-112 nmol/L) is used; however, the sensitivity is poor (~ 60%) [48]. Although higher cut-off values have been proposed, these have been associated with poorer specificity [49].

If an abnormal value is noted, the low-dose ACTH stimulation test should be performed to confirm the diagnosis. Given the poor sensitivity of the first morning cortisol measurement, a normal value does not rule out AS. Therefore, if the test result is normal, but the patient is experiencing symptoms suggestive of AS, a low-dose ACTH test is recommended.

Since cortisol levels decrease throughout the day, a random cortisol measurement is not an adequate measure of AS in children. Other measures of adrenal insufficiency are available, such as the assessment of urinary or salivary cortisol levels; however, these tests have not been well-studied in children with AS [50-52].

## Inhaled Corticosteroids (ICS): Pharmacokinetic and Pharmacodynamic Differences and Drug Interactions

Although the various ICSs available for the treatment of asthma are believed to have similar clinical efficacy when used at equivalent therapeutic doses, significant differences in their pharmacokinetics (PK) and pharmacodynamics (PD) exist which can impact their respective safety profiles. These differences warrant careful consideration when determining the benefits and risks of each ICS medication in an individual patient, particularly as they relate to the risk of systemic side effects such as AS [53]. Table 3 provides an overview of the PK and PD parameters that influence the safety of ICSs, such as oral bioavailability, lung deposition, protein-binding, half-life and systemic clearance [54].

**Table 3:** Pharmacodynamic (PD) and pharmacokinetic (PK) properties of the ICSs available for the management of asthma in Canada[53,54]

ICS	Oral bioavailability (%)	Lung deposition (%)	Particle size (μm)	Protein-binding (% not bound)	Half-life (h)	Systemic clearance (L/h)
Beclomethasone dipropionate	20/40*	50-60	<2.0	13	2.7*	150/120*

Budesonide	11	15-30	>2.5	12	2.0	84

Ciclesonide	<1/<1*	50	<2.0	1/1*	0.5/4.8*	152/228*

Fluticasone propionate	≤1	20	2.8	10	14.4	66

*active metabolite

**Figure 1:**
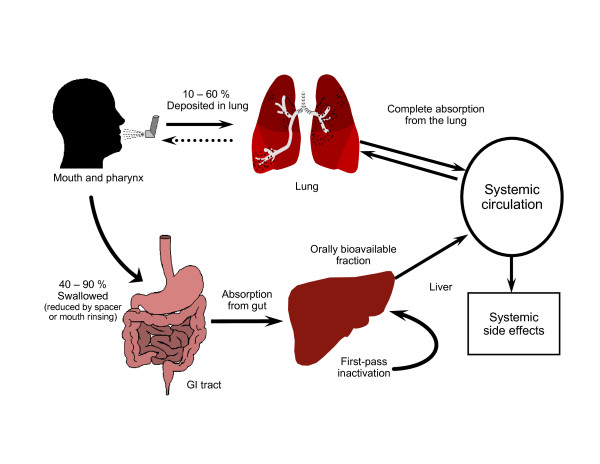
**Schematic representation of the fate of an ICS**. [53,58] Adapted from Derendorf et al., 2006 [53]; Derendorf, 1997 [58].

In order to better understand the effect of PK and PD parameters on safety, it is helpful to briefly review the fate of an ICS (see Figure 1). Depending on the inhaler device, approximately 10-60% of the administered ICS is deposited into the lungs upon inhalation. In the lungs, the ICS exerts its effect on inflamed tissue as soon as it dissolves into the pulmonary lining and binds to intracellular corticosteroid receptors. The remainder of the drug that does not get absorbed into the lung (40-90%) is deposited into the mouth and pharynx, where it has the potential to exert local side effects, such as oropharyngeal candidiasis and dysphonia. If not rinsed out of the mouth, this portion of the ICS dose may be swallowed and subsequently absorbed into the gastrointestinal (GI) tract (note that the amount swallowed can be reduced to as little as 10% through the use of a spacer) [55,56]. Drug that is absorbed from the GI tract and that escapes inactivation by first-pass metabolism in the liver enters the systemic circulation unchanged, potentially causing serious systemic side effects [53,57,58].

### Oral Bioavailability

The oral bioavailability of an ICS refers to the portion of the inhaled dose that is swallowed, escapes first-pass metabolism in the liver, and is available for systemic absorption (see Figure 1). Since the proportion of the ICS dose that is absorbed orally increases the potential for systemic side effects, it is advantageous for the oral bioavailability of an ICS to be relatively low. The oral bioavailability of the currently available ICSs varies widely, from approximately <1% for ciclesonide and fluticasone to 20-40% for beclomethasone (see Table 3) [53,57,59-61].

### Lung Deposition

Lung deposition refers to the amount of drug that enters the lung and exerts an effect at the site of inflammation. For ICSs to exert their optimal anti-inflammatory effect, a high lung deposition is generally desirable. Several factors impact pulmonary deposition including: (1) the physical properties of the ICS; (2) the delivery device; (3) particle size (discussed later); and (4) patient characteristics such as inhaler technique, age, and asthma severity [57,60]. As seen in Table 3, lung deposition is greatest with ciclesonide and beclomethasone [54].

### Particle Size

Particle size is an important determinant of the proportion of ICS that is deposited in the lower airways relative to the oropharyngeal cavity. Ideally, to be deposited in the bronchi and bronchioles, particles should be between 1-5 μm. Larger particles (>5 μm) are likely to be deposited into the oropharynx, while very small particles (<1 μm) will either be deposited in the upper airways or, if drawn into the lower airways, will be exhaled. Beclomethasone and ciclesonide delivered by metered-dose inhaler (MDI) have the smallest particle sizes among the available ICS medications [53,59].

### Prodrugs

Ciclesonide and beclomethasone are prodrugs that are inhaled as inactive compounds and then converted into their active metabolites (des-ciclesonide and 17-monopropionate, respectively) by enzymes located in the pulmonary epithelium [53,59]. Because prodrugs are inactive until they reach the lung, they are believed to be associated with fewer local side effects compared to ICSs that are administered in their active form (e.g., fluticasone and budesonide). In studies of ciclesonide, bioactivation within the oropharynx was shown to be very low, resulting in lower amounts of active drug in the oropharyngeal region compared with budesonide and fluticasone [62,63].

### Plasma Protein-Binding

When an ICS binds to plasma protein (albumin) in the systemic circulation, it is rendered pharmacologically inactive. Therefore, a high degree of plasma protein-binding is desirable to reduce the potential for systemic side effects [53,57,59,60]. Protein binding levels of budesonide, fluticasone and the active metabolite of ciclesonide (des-ciclesonide) have been shown to inversely correlate with degrees of cortisol suppression [64]. Both ciclesonide and des-ciclesonide are highly protein-bound (99%) in the systemic circulation (see Table 3) and have been shown to result in minimal suppression of cortisol [65].

### Half-life and Systemic Clearance

The metabolism and excretion of an ICS are factors contributing to the potential for systemic side effects. A short-half life and high clearance rate reduce the exposure of the ICS to the systemic circulation, thereby improving the safety profile. The elimination half-lives of the currently available ICSs range from 14.4 hours with fluticasone to as little as 0.5 hours with des-ciclesonide (Table 3) [54].

After systemic absorption, ICSs are metabolized primarily by the liver, and the clearance rate of most ICS medications is typically similar to or somewhat lower than the rate of hepatic blood flow (~90 L/h) (Table 3) [53,57]. However, the systemic clearance of the active metabolite of ciclesonide considerably exceeds hepatic blood flow, indicating that additional mechanisms of clearance by other organs are likely involved [59].

### Drug Interactions

In addition to the PK and PD properties of ICS therapies, it is important to note that clinically significant drug interactions have been noted with a number of ICS medications and potent inhibitors of cytochrome (CYP) 3A4 isozymes, such as ritonavir, ketoconazole, and itraconazole (Table 4 provides a list of the more potent CYP3A4 inhibitors). Concomitant administration of itraconazole and budesonide, for example, has been associated with a more than 4-fold increase in plasma concentrations of budesonide given by inhalation. The concomitant use of ritonavir and fluticasone has also been shown to greatly increase plasma fluticasone concentrations, leading to cases of Cushing's syndrome and AS. Approximately 25 cases (15 adult and 10 pediatric) of significant AS secondary to an interaction between ritonavir and ICSs have been noted, and most (24 of 25) have occurred with fluticasone. The vast majority of these cases were receiving high ICS doses prior to beginning the inhibitor, and Cushingoid appearances were often noted within 2 weeks of starting ritonavir therapy [66-70]. In 2004, the Health Products and Food Branch of Health Canada posted a public health advisory warning patients and healthcare professionals of this serious drug interaction, and advised that the concomitant use of fluticasone and ritonavir be avoided, unless the benefit to the patient outweighs the risk of systemic corticosteroid side effects [71]. If ritonavir is required, another ICS, such as low-dose budesonide or beclomethasone, should be considered and used with caution [72]. Clinicians should also consider the use of lower ICS doses with coadministration of other CYP3A4 inhibitors (see Table 4) [54].

**Table 4:** Examples of potent CYP3A4 inhibitors

Antibiotics	Quinupristin (Synercid)
**Antidepressants**	Fluvoxamine (Luvox) Nefazodone (Serzone)

**Antifungal agents**	Fluconazole (Diflucan) Itraconazole (Sporanox) Ketoconazole (Nizoral) Voriconazole (Vfend)

**HIV Drugs**	Amprenavir (Agenerase) Atazanavir (Reyataz) Delavirdine (Rescriptor) Indinavir (Crixivan) Nelfinavir (Viracept) Ritonavir (Norvir) Saquinavir (Invirase)

**Miscellaneous**	Cyclosporine (Neoral)

HIV: human immunodeficiency virus

## Effects of ICS Therapy on Adrenal Suppression (AS): Review of the Evidence

### Biochemical Evidence

Biochemical evidence of AS, which is often assessed using an early morning cortisol measurement or the low-dose ACTH stimulation test, is commonly found in children receiving ICS therapy, particularly those using high doses [25]. However, a high degree of inter-individual susceptibility to AS has been noted and is likely related to individual patient factors including asthma severity. Biochemical evidence of AS is most commonly seen with fluticasone, particularly at doses ≥500 μg/day. In a study of 18 asthmatic children treated with approximately 500 μg/day of fluticasone, half of the subjects studied had biochemical evidence of AS (as assessed by the insulin tolerance test) up to 16 weeks after starting fluticasone therapy [73]. Similar findings were noted in a study of 34 children on high-dose ICS therapy who were switched to either fluticasone 750 μg/day or beclomethasone 1500 μg/day. Twelve weeks after the switch, abnormal low-dose ACTH test results indicative of AS were noted in over 60% of patients in each treatment group [74]. Paton et al. used the low-dose ACTH stimulation test to assess adrenal function in 194 children receiving ≥500 μg/day of fluticasone and found that approximately 40% of these subjects had evidence of AS [18].

One study of 16 healthy adults found doses of beclomethasone ≥1000 μg/day to be associated with significant suppression of overnight urinary cortisol-to-creatine ratio [75]. A 12-month observational study of 35 asthmatic children (aged 4-10 years) using ≥1,000 μg/day of budesonide (median = 1,600 μg/day) or equivalent doses of fluticasone (median = 1000 μg/day) for at least 6 months, found biochemical evidence of AS (using the low-dose ACTH stimulation test) in 46% of subjects [35]. However, AS appears to be rare on budesonide doses of <400 μg/day, even with long-term treatment. One study found no change in HPA-axis function, as measured by basal cortisol levels, in children who received open-label budesonide at a dose of 400 μg/day for 12 months [76]. Bacharier et al. also found that long-term (3-year) treatment with budesonide 400 μg/day had no significant effect on HPA-axis function (as measured by standard-dose ACTH stimulation testing and urinary cortisol excretion) in children with mild-to-moderate asthma [77].

Few good-quality studies have compared the frequency of AS among the various ICS formulations. In a Cochrane review comparing beclomethasone, budesonide and fluticasone, investigators were unable to make any conclusions regarding the comparative safety of these agents given the lack of available data [78]. However, other studies and meta-analyses have suggested that there is increased suppression with high-dose fluticasone compared with other medications at equivalent doses. A meta-analysis examining the systemic adverse effects of fluticasone, budesonide, and beclomethasone found marked biochemical evidence of AS at high ICS doses (>750 μg/day of fluticasone, >1500 μg/day of budesonide/beclomethasone). Fluticasone was found to exhibit greater dose-related AS than the other ICS therapies studied, particularly at doses above 800 μg/day. The investigators concluded that this finding may be due to the specific PK properties of fluticasone [79].

Unlike other ICS medications, ciclesonide appears to have little or no suppressive effects on the HPA axis [80]. Clinical studies of ciclesonide administered at doses up to 640 μg/day have failed to show any significant effects of this ICS on serum or 24-hour urinary cortisol levels [81-83]. In a 12-week, double-blind, randomized, placebo-controlled study comparing ciclesonide 320 μg/day OD or BID and 440 μg/day of fluticasone TID to placebo, ciclesonide was associated with peak cortisol levels (as assessed by both low- and high-dose ACTH stimulation tests) and 24-hour urinary free cortisol levels that were similar to those noted with placebo. Fluticasone, on the other hand, was associated with significant reductions in serum cortisol levels and 24-hour urinary free cortisol levels compared to placebo [84]. In another study of 4 asthmatic children with AS due to the use of inhaled fluticasone, normalization of HPA-axis function was found after subjects were switched to ciclesonide [23].

### Clinical Evidence

With more than 60 case reports published in the literature, there is now strong clinical evidence supporting the presence of ICS-associated AS [22]. The majority of these cases have presented with signs of adrenal crisis, particularly altered consciousness (seizure, coma) secondary to hypoglycemia, and had evidence of HPA-axis suppression on testing. There are also case reports of AS without crisis, including poor weight gain, poor linear growth or other non-specific symptoms. Almost all cases have involved children being treated with ≥500 μg/day of fluticasone [17,26,85-88].

The largest series of case reports comes from a national survey conducted in the UK, which identified 33 cases of AS (28 children and 5 adults). AS was confirmed with an ACTH stimulation test in the large majority of cases (29) and by other measures of adrenal function in the remaining 4 cases. All cases were treated with high ICS doses (500-2000 μg/day). Despite the fact that the vast majority with AS (91%) were receiving fluticasone, only 16% of all patients were using fluticasone in the UK at the time of this study. All but five children presented with acute hypoglycemia characterized by seizure, decreased levels of consciousness or coma; 1 death was noted. The investigators concluded that the frequency of acute adrenal crisis was greater than expected, particularly in fluticasone-treated children [17].

Apart from exposure to illness or surgery, risk factors for the development of adrenal crisis in children with AS are not well understood. Therefore, increased awareness and early recognition of AS are important to help prevent against this potentially serious consequence of ICS therapy.

## Recommendations for the Prevention, Screening and Management of Adrenal Suppression (AS)

As highlighted previously, AS often goes unrecognized until physiologic stress precipitates an adrenal crisis. As a result, the frequency of AS in the asthma population is not well documented. Currently, there are no national guidelines for AS screening in children with asthma. Evidence suggests that screening approaches vary widely, and that many children with asthma who are at risk for AS are not screened. Guidelines in the UK state that the use of fluticasone at doses ≥400 μg/day should be accompanied with screening for AS [89]. Brodlie and McKean investigated the screening practices of 14 tertiary pediatric respiratory centres in the UK and found that, despite these guidelines, less than 60% had an official policy for screening children with asthma. The investigators also found significant differences in the threshold ICS dose used to start testing for AS, the type of screening tests performed, and the interpretation of test findings. In children prescribed fluticasone, 50% of centres tested for AS at ≥500 μg/day, 21% at ≥1000 μg/day and, in 29%, the cut-off dose for testing varied. For beclomethasone, 50% of centres tested at ≥1000 μg/day, 14% at ≥1500-2000 mg/day and, in 36% of centres, various cut-off doses for testing were used. When considering AS testing, the use of oral prednisolone was taken into consideration by less than 60% of centres. A low-dose ACTH stimulation test was performed in 50% of centres, a high-dose test in 21%, and a morning cortisol measurement in only 8%; in 21% of centres, the screening test used varied. In total, only 57% of respondents regarded AS as a significant problem [90].

Given these findings as well as the lack of published guidelines for the prevention, screening and management of AS, the authors have provided the recommendations in this section based on the best available literature and expert opinion.

### Prevention

Although ICS therapy represents the mainstay of asthma management, physicians and other healthcare professionals need to be aware of the risk for AS in all asthma patients using ICS therapy, regardless of the dose prescribed. Although most cases have been reported in individuals using high doses of fluticasone, a few cases have also been noted in patients using low ICS doses [23,27,91].

Recognizing children at risk is imperative, and screening should be considered in any child with symptoms of AS, children using high ICS doses, or those with a history of oral corticosteroid use (see Screening section for more detail). Although poor growth is not always indicative of AS, growth should be monitored every 6 months (ideally by using stadiometry measurements) and measurements should be plotted on an appropriate growth curve. If, after 6 months, growth velocity appears to be inadequate, the physician should consider all possible etiologies, including AS, as well as referral to an endocrinologist for appropriate testing.

Healthcare professionals should educate the parents of children using high ICS doses about the risk of AS and associated symptoms (see Table 2). Emergency contact information should be provided to parents and caregivers in the event of severe symptoms or suspected adrenal crisis.

Most cases of adrenal crisis resulting from ICS therapy have been associated with poor patient follow-up and inappropriately high ICS doses [27]. Therefore, physicians should regularly re-evaluate the child's ICS dose to ensure the lowest effective dose is utilized to control asthma symptoms. Physicians should also consider clinically important differences between ICS medications (see section on PK and PD properties of ICS) as well as the patient's total steroid load (i.e., consider the use of all forms of glucocorticoid therapy including oral, inhaled, intranasal, intramuscular and intravenous). In patients at risk of AS, consideration should be given to the use of an ICS with minimal adrenal effects and the best benefit-to-side effect ratio.

### Screening

Screening is recommended in all children presenting with symptoms of AS (including poor growth), regardless of the ICS dose utilized (see Table 5). It should also be considered in: (1) asymptomatic patients who have been treated for 3-6 months with ≥500 μg/day of fluticasone or ≥1000 μg/day of budesonide/beclomethasone; (2) children who have received a course of oral corticosteroids for more than 2 consecutive weeks; and (3) children who have received multiple courses of oral steroids amounting to more than 3 weeks in the last 6 months [24,27,53,61]. Although there is no evidence and no case reports of AS with ciclesonide use, the authors feel the safest approach at the present time is to screen patients using over 1000 μg/day for 3-6 months. The authors also recommend screening all children using concomitant ICS therapy and antiretroviral or antifungal agents (e.g., itraconazole, ketoconazole, voriconazole) since these potent CYP3A4 inhibitors can potentiate the systemic side effects of ICS treatment.

**Table 5:** Screening recommendations for AS

When to Screen?	► Patient has persistent symptoms of AS: Weakness/fatigue, malaise, nausea, vomiting, diarrhea, abdominal pain, headache (usually in the morning), poor weight gain, myalgia, arthralgia, psychiatric symptom, poor growth, hypotension*, hypoglycemia* ► Patient has been receiving high-dose ICS therapy for 3-6 months: ≥500 μg/day of fluticasone; ≥1000 μg/day of budesonide/beclomethasone; or >1000 μg/day of ciclesonide ► Patient has received oral corticosteroids for: >2 consecutive weeks or >3 cumulative weeks in the last 6 months ► Patient using concomitant ICS therapy and potent CYP3A4 inhibitors, particularly antiretroviral and antifungal agents
	► Complete first morning (08:00 am) cortisol test
	- Must be completed by 8:00 am or earlier
	- No oral glucocorticoids the evening and morning prior to the test
	- Fasting not required
**How to Screen?**	► If result is normal, screen again in 6 months
	► If result is normal but patient has symptoms of AS, perform low-dose ACTH stimulation test to confirm diagnosis:
	- 1 μg of cosyntropin; cortisol levels taken at 0, 15-20 and 30 minutes
	- Peak cortisol < 500 nmol/L = AS (peak >500 nmol/L is normal)

**When to be Concerned?**	► 8:00 am cortisol value < 85 nmol/L = diagnosis of AS ► 8:00 am cortisol value < laboratory normal = possible AS; consider endocrinology referral for confirmation of diagnosis

AS: adrenal suppression; ICS: inhaled corticosteroid

*Symptoms of adrenal crisis require emergent management

Given the ease and practicality of a first morning cortisol measurement, it should be considered for the initial screening of these patients. The test should be performed at 8:00 am or earlier given that cortisol levels decline throughout the day with natural circadian rhythm. For children receiving oral glucocorticoids, both the evening and morning glucocorticoid doses should be held prior to testing (see Table 5).

If the 8:00 am cortisol value is below the laboratory normal, AS should be suspected and a referral to a pediatric endocrinologist should be considered for confirmation of the diagnosis using the low-dose ACTH test. A very low morning cortisol level (i.e., <85-112 nmol/L) is diagnostic of AS and warrants an urgent referral to a pediatric endocrinologist. If the result is within the laboratory normal range, the child should be screened every 6 months. It is important to remember that the sensitivity of a first morning cortisol measurement is poor and, therefore, a normal value does not rule out the presence of AS. If the child has a normal test result, but symptoms of AS are present, a low-dose ACTH stimulation test should be performed to confirm the diagnosis.

### Management

Whenever possible, cases of AS should be managed in consultation with a pediatric endocrinologist. Daily hydrocortisone at a physiologic dose (8-10 mg/m^2^/day) should be considered until the first morning cortisol value normalizes (see Table 6). Dosing and timing of daily glucocorticoid replacement should be discussed with an endocrinologist. In all patients with proven AS, stress steroid dosing (high doses of hydrocortisone) during times of illness or surgery must be provided to simulate the protective endogenous elevations in cortisol levels that occur with physiological stress. For mild-to-moderate illness, 20-30 mg/m^2^/day of hydrocortisone, divided BID or TID, is recommended. For adrenal crisis, a cortisol level should be drawn immediately (to prove suppression), the endocrinologist on call should be contacted, and the child should be treated with an immediate stress dose of intravenous or intramuscular hydrocortisone (100 mg/m^2^), followed by 100 mg/m^2^/day of hydrocortisone, divided into 3 to 4 doses over a 24-hour period [22]. In patients on supraphysiological doses of oral corticosteroids for more than 2 consecutive weeks or those who have required more than 3 weeks of oral steroids over the course of 6 months, tapering of steroids should be considered to allow for adrenal recovery. In patients with proven AS, consideration should also be given to the use of ciclesonide, which has been shown to have little or no suppressive effects on the HPA axis [80].

**Table 6:** Recommendations for the management of AS

**1.**	**Stress steroids during periods of physiological stress**
	**- *Adrenal crisis: ***Hydrocortisone injection (Solu-Cortef) 100 mg/m^2 ^(max. 100 mg) IV/IM stat with saline volume expansion, followed by 25 mg/m^2 ^q 6 hours (max. 25 mg q 6 hours); call endocrinologist on call
	**- *Surgery: ***Hydrocortisone injection (Solu-Cortef) 50-100 mg/m^2 ^IV (max 100 mg) pre-operatively, then 25 mg/m^2 ^q 6 hours (max 25 mg q 6 hours); call endocrinologist on call
	**- *Illness or fever: ***20 mg/m^2^/day hydrocortisone equivalent, divided BID or TID
	**- *Fever >38.5***^***o***^***C or vomiting: ***30 mg/m^2^/day hydrocortisone equivalent, divided TID
	**- *Unable to tolerate orally: ***Hydrocortisone must be administered parenterally as Solu-Cortef, 25 mg/m^2^/dose q 6 hours IV or q 8 hours IM
**2.**	**± Daily physiologic dose of hydrocortisone (8-10 mg/m**^**2**^**/day)**
**3.**	**Family education**
	- Stress steroid dosing
	- Emergency medical contact information in case of illness
4.	**Information card/Medic-Alert bracelet**

IV: intravenous; IM: intramuscular; BID: twice daily; TID: three times daily; QID: four times daily; q: every

Parents and children at risk for AS should be educated about stress steroid dosing and provided with emergency medical contact information in the event of illness. Consideration should be given to providing patients with a Medic-alert bracelet and/or information card detailing their diagnosis, updated medication doses and stress-dosing instructions.

## Conclusions

In spite of the measurable effects of ICS therapy on the HPA axis, it is important to remember that effective anti-inflammatory therapy is essential for the treatment of asthma, that ICSs are the most effective anti-inflammatory agents available, and that the suppressive effects of ICS therapy on the HPA axis is markedly less than clinically equivalent doses of oral corticosteroids. At low-to-moderate doses, ICS therapy does not present any significant risk for systemic side effects. However, when high doses are used for prolonged periods, serious adverse events, including AS, are possible. The risk for AS can be minimized through increased awareness and early recognition of at-risk patients, regular patient follow-up to ensure the lowest effective ICS doses are utilized, and by choosing an ICS medication with minimal systemic effects. When high-dose ICS therapy is required, important differences in the PK and PD characteristics of the available ICSs warrant consideration in clinical practice. For patients with proven AS, family education and stress steroids during times of illness, injury or surgery are imperative and will help reduce the morbidity associated with this serious complication of ICS therapy.

## Competing interests

Dr. Alexandra Ahmet has received honoraria for continuing education from Nycomed, MD Briefcase and Peer Review.

Dr. Harold Kim is the past president of the Canadian Network for Respiratory Care and co-chief editor of *Allergy, Asthma and Clinical Immunology*. He has received consulting fees and honoraria for continuing education from AstraZeneca, GlaxoSmithKline, Graceway Pharmaceuticals, King Pharma, Merck Frosst, Novartis, and Nycomed.

Dr. Sheldon Spier has received consulting fees and honoraria for continuing medical education from AstraZeneca, Graceway Pharmaceuticals, Merck Frosst and Nycomed.

## Authors' contributions

AA contributed to the conception, drafting and writing of the manuscript and to revising it for important intellectual content. HK and SS contributed to the drafting and development of the manuscript and to revising it critically for important intellectual content. All authors read and approved the final manuscript.
